# An improved CS-LSSVM algorithm-based fault pattern recognition of ship power equipments

**DOI:** 10.1371/journal.pone.0171246

**Published:** 2017-02-09

**Authors:** Yifei Yang, Minjia Tan, Yuewei Dai

**Affiliations:** 1 School of Automation, Nanjing University of Science and Technology, Nanjing, Jiangsu, China; 2 School of Electronics and Information, Jiangsu University of Science and Technology, Zhenjiang, Jiangsu, China; Nanjing Normal University, CHINA

## Abstract

A ship power equipments’ fault monitoring signal usually provides few samples and the data’s feature is non-linear in practical situation. This paper adopts the method of the least squares support vector machine (LSSVM) to deal with the problem of fault pattern identification in the case of small sample data. Meanwhile, in order to avoid involving a local extremum and poor convergence precision which are induced by optimizing the kernel function parameter and penalty factor of LSSVM, an improved Cuckoo Search (CS) algorithm is proposed for the purpose of parameter optimization. Based on the dynamic adaptive strategy, the newly proposed algorithm improves the recognition probability and the searching step length, which can effectively solve the problems of slow searching speed and low calculation accuracy of the CS algorithm. A benchmark example demonstrates that the CS-LSSVM algorithm can accurately and effectively identify the fault pattern types of ship power equipments.

## 1. Introduction

Ship power equipments often work under terrible working conditions. Thus, the occurrence of faults is unavoidable. It should be mentioned that serious faults can lead to economic losses and threaten the safety of the crew's life. However, the structure of ship power equipments is complex, and it is difficult to figure out the fault pattern. Then, it is necessary to analyze the fault pattern of ship power equipments and guarantee the reliability and safety of ship power equipments accordingly.

Ship power equipments include diesel, gearbox, air compressor, pump, etc. The fault pattern recognition for these equipments falls into a multi-classification issue which focuses on the fault samples. As observed from [1], neural network demonstrates the characteristics of nonlinear processing, adaption, robustness, etc. Then it can be widely used to deal with nonlinear equipment test signal, complex fault modes, and fault recognition. However, this method does show some shortcomings such as the complex network structure, the occurrence of local extremum, and slow converging speed [2].

Some new algorithms, especially the SSVM in [3] and the CBoPS in [4], are proposed in [3–8] to enhance the searching accuracy and searching speed. Moreover, the proposed algorithms find applications in mobile visual search [4], mobile visual location recognition (MVLR) [5–7], and mobile landmark recognition [8].

The least squares support vector machine (LSSVM), which is a fusion of neural network and support vector machine (SVM), is an extension of SVM [9]. By using equality constraints to substitute inequality constraints and solving linear equations to obtain the support vector, one can simplify the calculation and enhance the training speed [10].

The parameter optimization of LSSVM can affect greatly the prediction model performance of LSSVM. Considering that the optimization of LSSVM parameters is necessary, this paper adopts the Cuckoo search algorithm (CS).

## 2. Description of the LSSVM method

The LSSVM method introduces the idea of square sum of errors to the objective function of the standard SVM, where a training data with *n* samples is taken into account.

*Train* = {(*x*_*i*_,*y*_*i*_)|*i* = 1,2,⋯,*n*}, where the input data is *x*_*i*_ ∈ *R*^*d*^, and the output characteristic is *y*_*i*_ ∈ *R*. Utilize a nonlinear mapping *φ*(⋅) to project the sample input space to the feature space *φ*(*x*) = {*φ*(*x*_1_),⋯,*φ*(*x*_*n*_)}, and construct the following optimal linear decision function [11]

$$y(x)=\omega^{T}\cdot \varphi (x)+\beta$$ (1)

By using the structural risk formula

$$R=\frac{1}{2}{\left\|\omega \right\|}^{2}+C\cdot R_{emp}$$ (2)

one can calculate the weight vector *ω* and the offset *β* in Eq (1). In Eq (2), *C* and *R*_*emp*_ denote the penalty factor and the loss function, respectively. In LSSVM, the loss function is always equal to the quadratic loss function $R_{emp}=\sum_{i}\xi_{i}^{2}$, where *ξ*_*i*_ denotes the degree that the mis-classification sample deviates from the ideal sample. Then, getting the solutions of *ω* and *β* is equivalent to solving the following optimization problem

$$\begin{array}{l} \min R=\frac{1}{2}\cdot {\left\|\omega \right\|}^{2}+C\cdot \sum_{i=1}^{n}\xi_{i}^{2}\\ s.t.\;y_{i}=\omega^{T}\cdot \varphi (x_{i})+\beta +\xi_{i},\;i=1,\cdots ,n \end{array}$$ (3)

Introduce the Lagrangian multiplier *α* = {*α*_1_,*α*_2_,⋯,*α*_*i*_} to formula (3) and construct an equation as

$$L(\omega ,\beta ,\xi_{i},\alpha )=\frac{1}{2}\cdot {\left\|\omega \right\|}^{2}+C\cdot \sum_{i=1}^{n}\xi_{i}^{2}-\sum_{i=1}^{n}\left(\alpha_{i}\cdot (\omega^{T}\cdot \varphi (x_{i})+\beta +\xi_{i}-y_{i})\right)$$ (4)

Calculate the derivative of every factor in the formula (4). For the purpose of eliminating *ω* and *ξ*, introduce the equations $\omega =\sum_{i=1}^{n}\alpha_{i}\varphi (x_{i})$ and 2*C*⋅*ξ*_*i*_ = *α*_*i*_ to (4). Then one can obtain a new formula described as

$$y_{i}=\sum_{j=1}^{n}(\alpha_{j}\cdot <\varphi (x_{j}),\varphi (x_{i})>)+\beta +\frac{\alpha_{i}}{2C}$$ (5)

Suppose that there exists a kernel function *K*(*x*_*i*_,*x*_*j*_) = <*φ*(*x*_*j*_),*φ*(*x*_*i*_)> which satisfies the Mecer condition [12]. Then the formula (5) can be described as

$$y_{i}=\sum_{j=1}^{n}(\alpha_{j}\cdot K(x_{i},x_{j}))+\beta +\frac{\alpha_{i}}{2C}$$ (6)

Solve the formula (6) to get the model parameters *β* and [*α*_1_,⋯,*α*_*n*_]^*T*^. Then the decision function of LSSVM is shown as (see also [13])

$$y(x)=\text{sgn}[\sum_{i=1}^{n}\left(\alpha_{j}\cdot K(x,x_{i}))+\beta \right]$$ (7)

It is well-known that different types of kernel function *K*(*x*_*i*_,*x*_*j*_) will lead to different performance of LSSVM, while the polynomial kernel function, the sigmoid kernel function, the linear kernel function and the radial basic function (RBF) are widely used now. The experiment in [14] illustrates that the RBF is more general than other functions mentioned above. The RBF is usually described as

$$K(x,x_{i})=\exp(-\frac{{\left|x-x_{i}\right|}^{2}}{2\sigma^{2}})$$ (8)

Thus we choose the formula (8) as the kernel function for constructing the LSSVM classifier of ship power equipments’ fault. However, the factor *σ* in the kernel function and the penalty factor *C* should be optimized. The improved Cuckoo Search (CS) algorithm is proposed in this paper to optimize the system performance.

## 3. The improved CS algorithm

### 3.1 Description of the standard CS algorithm

The main mechanism of the CS algorithm is based on the strategy of cuckoo’s population with parasitical multiplying. This algorithm is to find out the optimal egg through its Lévy flight, which approach can achieve a high efficiency optimization [15] with one egg representing one optimal solution. The nest host will abandon its own nest once an outside egg is discovered, and then rebuild the nest in another place, where the new nest represents the new optimal solution. Therefore, the main idea of the CS algorithm is to adopt new and good solutions to substitute bad solutions [16]. There are two methods to signify the process of producing offspring.

#### 3.1.1 The Lévy flight

If the Lévy flight [15] is introduced, the path of seeking nest and the expression of location updating can be described as

$$x_{i}^{k+1}=x_{i}^{k}+\alpha \circ L(\lambda ),\;i=1,2,\cdots ,n$$ (9)

where $x_{i}^{k}$ represents the location of the *i*th nest in the *k*th generation, ∘ denotes Hadamard product, *α* is the degree of step control, *L*(*λ*) is a random searching path which satisfies Lévy flight distribution, while *s* is the random step length satisfying

$$L(s,\lambda )\sim s^{-\lambda },(1<\lambda \leq 3)$$ (10)

Adopt a formula about the path of Lévy flight which is proposed by Mantegna to describe this distribution (see also [17])

$$L(\lambda )={\mu /\left|v\right|}^{1/\beta },0<\beta \leq 2$$ (11)

where *β* = *λ*−1 with *β* being usually chosen as *β* = 1.5. The parameters *μ*, *ν* satisfy a normal distribution.

#### 3.1.2 Deviation of random walking

Under some cases, the nest host will probably discover the eggs of cuckoos. Define the probability of the nest host discovering the cuckoo egg as *P*_*a*_. Then one has

$$x_{i}^{k+1}=x_{i}^{k}+r\cdot (x_{j}^{k}-x_{t}^{k})⊗H(P_{a}-r)$$ (12)

where *x*_*i*_, *x*_*j*_ *x*_*t*_ are randomly chosen different solutions, *H*(⋅) is a Heaviside function and *r* is a random number which satisfies the (0,1) uniform distribution.

### 3.2 The improved CS algorithm

#### 3.2.1 The improvement of the recognition probability

When the nest location is updated, if *r* > *P*_*a*_, the nest location $x_{i}^{k+1}$ will be updated, otherwise, it will keep unchanged. Then, the smaller the *P*_*a*_, the larger the probability of discovering outside eggs, and the number of updated nests will increase. One should choose an appropriate *P*_*a*_ to achieve a balance between the influences of the global extremum and the local extremum.

As observed from [18], if the probability falls into the interval [0.1, 0.75], the global search capability will increase with the increase of the number of iterations. Introduce a dynamic adaptive mechanism to improve the probability within an appropriate range

$$P_{a,i}^{k}=P_{a\min}+(P_{a\max}-P_{a\min})(1-\frac{f_{best}^{k}}{f_{i}^{k}})$$ (13)

where $P_{a,i}^{k}$ represents the probability of the *i*th nest host finding outside eggs in the *k*th generation. Similarly, *P*_*a*max_ and *P*_*a*min_ represent the bounds of maximum and minimum probabilities of recognition. At the *k*th generation population, $f_{best}^{k}$ represents the best individual and $f_{i}^{k}$ represents the *i*th individual fitness.

Note that if $f_{i}^{k}=f_{best}^{k}$, the computation process will terminate. Thus, the value of *P*_*a*_ in formula (13) will increase with nests fitness values getting close to each other. Since the adaptive strategy of the algorithm is fully realized, the CS algorithm can achieve a balance between the global random searching ability and the local searching ability.

#### 3.2.2 The improvement of adaptive step

In the Standard CS algorithm, the Lévy flight is adopted to generate a randomly variable step length. For the searching process, a large step length will lead to the increase of the probability of getting the global optimum. However, the searching precision will decrease and the phenomenon of oscillation is unavoidable. If a small step length is adopted, the searching precision is increased at the cost of reduced searching speed.

To reduce the influence of the Lévy flight randomly determined searching step length, and deal with the relationship between the ability of global optimization and the accuracy of optimization, we introduce the following equation (see also [19])

$$d_{i}=\frac{\left\|n_{i}-n_{best}\right\|}{d_{\max}}$$ (14)

Based on formula (14), one can propose an adaptive strategy to renew the step length which is described as

$$step_{i}=step_{\min}+(step_{\max}-step_{\min})d_{i}$$ (15)

In the above given formula, *n*_*i*_ represents the *i*th position of the nest, *n*_*best*_ represents the best nest corresponding to the best position, *d*_max_ represents a maximum value of the distance between the optimal nest location and the others, while *step*_max_ and *step*_min_ represent the maximum and the minimum searching step lengths.

The smaller the distance between the *i*th nest position and the best nest position, the smaller the step length, otherwise, the larger the mentioned distance, the larger the step length. Then, the step length of the current iteration can be updated dynamically via the result of the last iteration, which demonstrates a good adaptivity. Then one can choose *α* = *step*_*i*_, and adopt formula (9) to calculate the path of seeking nest and the nest location update.

### 3.3 Performance analysis of improved CS algorithm

**Fig 1:**
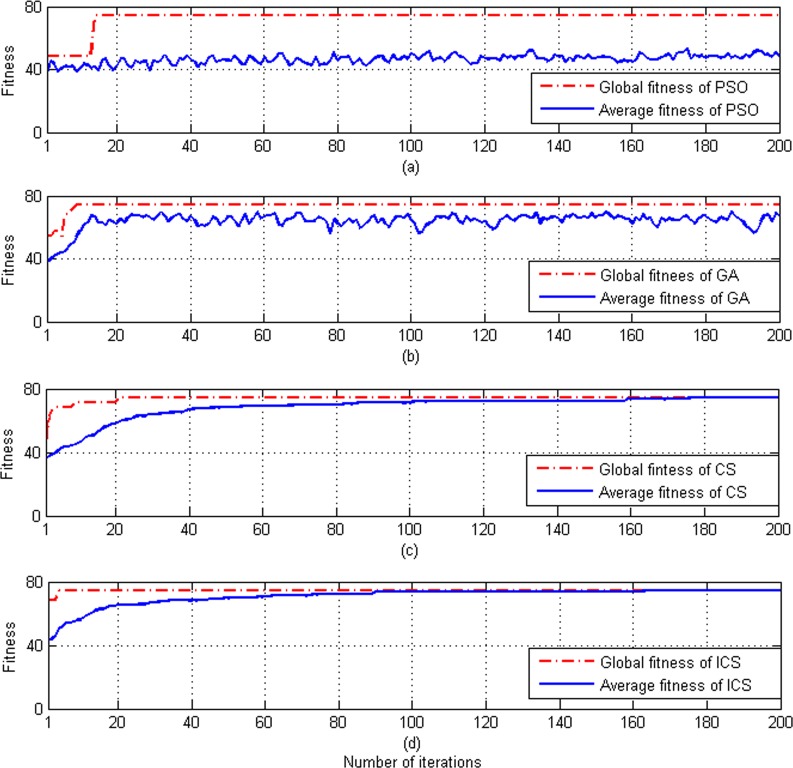
Optimization parameters of the PSO method, the GA method, the CS method and the improved CS method. (a) Curve of fitness (PSO method) with c1 = 1.5, c2 = 1.7, and number of the populations = 20. (b) Curve of fitness (GA method) with number of the populations = 20. (c) Curve of fitness (CS method) with number of the nests = 20. (d) Curve of fitness (ICS method) with number of the nests = 20.

To verify the effectiveness of the improved CS algorithm, we compare the performance between the improved CS (ICS) algorithm and the existing particle swarm optimization (PSO), genetic algorithm (GA), and standard CS algorithm. The parameter optimization results of the PSO method, the GA method, the standard CS method, and the ICS method are presented in Fig 1(A), 1(B), 1(C) and 1(D), respectively.

As one can see from Fig 1, the improved CS method provides better fitness and higher optimization speed than other algorithms. Moreover, the average fitness of ICS is relatively stable.

**Fig 2:**
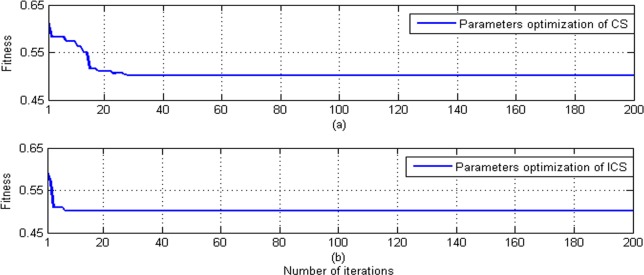
Convergence speeds of the CS method and the improved CS (ICS) method. (a) The convergence speed of the CS method. (b) The convergence speed of the ICS method.

Choosing the mean square error of test sample’s predictive value as fitness, one can obtain the convergence speeds of the standard CS method and the improved CS method, see Fig 2.

As one can see from Fig 2, the improved CS method provides a higher speed of optimization convergence than the standard CS method, which illustrates the merits of the newly proposed improved algorithm.

## 4. The improved CS-LSSVM algorithm-based fault pattern recognition

### 4.1 The process of fault pattern recognition

**Step 1:** In order to reduce the dimensionality, the equipment vibration signal data is collected and extracted from the kernel principal component by using the KPCA method for reducing the correlation between the characteristics of the data. The LSSVM training samples and testing samples are generated later.

**Step 2:** Initialize parameters which include the number of nests *n*, searching accuracy, the maximum number of iterations, the maximum and the minimum step length *step*_max_ and *step*_min_ of the CS algorithm. Generate initial locations of *n* nests randomly, which are defined as $p_{0}={\left[x_{1}^{\left(0\right)},x_{2}^{\left(0\right)},\cdots ,x_{n}^{\left(0\right)}\right]}^{T}$ here. Assume that the location of a nest can be defined as (*C*,*σ*). Then we can find out the best nest location $x_{b}^{\left(t\right)}$ and the best fitness $f_{best}^{\left(t\right)}$, where *t* = 0.

**Step 3:** Reserve the location of last generation’s nest as $x_{b}^{\left(t-1\right)}$. Calculate the step length of Lévy flight by using formula (14) and (15). Update the locations of *n* nests. For *n* nests, one can get the new locations from formula (12) and calculate the fitness $f_{n}^{\left(t\right)}$ by using LSSVM.

**Step 4:** Compare the newest locations with the last nests locations $p_{t-1}={\left[x_{1}^{\left(t-1\right)},x_{2}^{\left(t-1\right)},\cdots ,x_{n}^{\left(t-1\right)}\right]}^{T}$. Update them for getting better locations $p_{t}={\left[x_{1}^{\left(t\right)},x_{2}^{\left(t\right)},\cdots ,x_{n}^{\left(t\right)}\right]}^{T}$.

**Step 5:** Determine to reserve or change the locations of nests according to the adaptive recognition probability of formula (13). Generate a random number *r*, which is uniformly distributed, as the probability of nest host recognizing the outside eggs, and compare *r* with *P*_*a*_. Reserve the nest which is unidentified, in the meantime, change the nest location $x_{n}^{\left(t+1\right)}$ which can recognize the outside eggs according to formula (12). Get new locations of the nests $p_{t+1}={\left[x_{1}^{\left(t+1\right)},x_{2}^{\left(t+1\right)},\cdots ,x_{n}^{\left(t+1\right)}\right]}^{T}$.

**Step 6:** Compare *p*_*t*+1_ in **Step 5** with *p*_*t*_ in **Step 4**. Substitute the location in *p*_*t*_ with the location which provides better fitness in *p*_*t*+1_. Judge whether the best fitness $f_{\text{best}}^{\left(k\right)}$ meets the searching accuracy or not. If this condition is satisfied, go to **Step 7**, otherwise, go to **Step 3**.

**Step 7:** Adopt the best nest location $x_{b}^{\left(k\right)}$ and the corresponding value (*C*,*σ*) as the optimal parameters for LSSVM to recognize the fault pattern. Judge whether the recognition accuracy meets the requirement or not. If this condition is satisfied, output the result, otherwise, go to **Step 3**.

**Fig 3:**
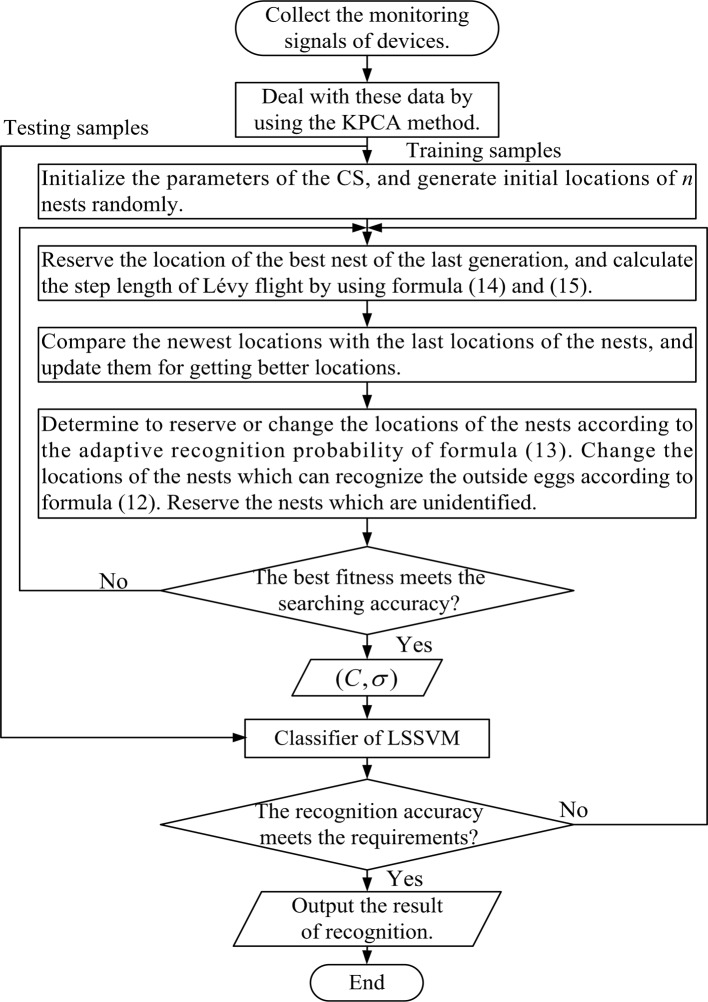
Flow chart of the improved CS-LSSVM-based fault pattern recognition.

The process of the improved CS-LSSVM-based fault pattern recognition is presented in Fig 3.

### 4.2 The case analysis of fault pattern recognition

The analysis of fault pattern recognition is based on a practical MTU diesel engine. The feasible fault patterns include that fuel delivery valve is worn (FW), fuel feeding is abnormal (FA), fuel supply advance is late (FL), fuel supply advance is early (FE), needle valve is stuck (NS), needle valve is worn (NW), and so on. The definition of different fault patterns is shown in Table 1.

**Table 1:** The definition of different fault patterns.

Fault label	Pattern	Fault label	Pattern
1	Normal	5	FE
2	FW	6	NS
3	FA	7	NW
4	FL	null	null

**Fig 4:**
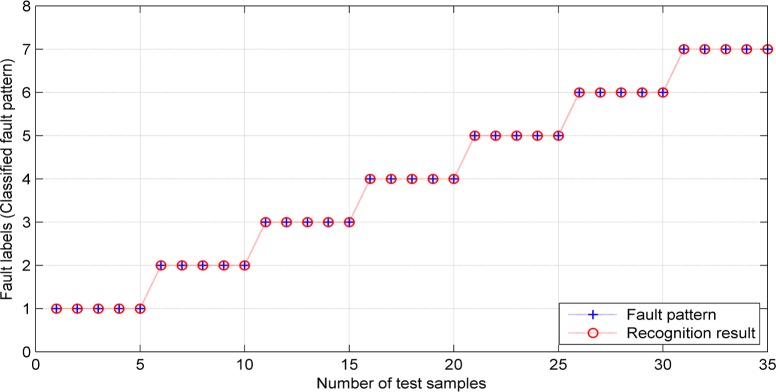
The improved CS-LSSVM-based recognition result.

The diesel engine vibration signal is collected under the sampling frequency 6000Hz. Then the KPCA method is adopted to reduce the dimensionality of itself for getting 8 characteristic values including peak-to-peak value *x*_*p−p*_, mean of absolute value ${\bar{x}}_{av}$, root-mean-square *x*_*rm*_, kurtosis *β*, wave index *s*_*f*_, peak index *C*_*f*_, impulse index *I*_*f*_, and margin index *CL*_*f*_ [20]. There are 7 different vibration signals. Every vibration signal includes 8 characteristic values. Every characteristic value includes 10 samples. Then the total number of sample data is 560. Choose 5 samples among them as training samples with the total number being equal to 35. The other 35 samples are test samples. Use the improved CS-LSSVM algorithm to deal with these samples. Because of the randomness of the meta-heuristic algorithm, we take 200 iterations here. By using the improved CS-LSSVM, we can get *C* and *σ*. The corresponding recognition result is shown in Fig 4.

As one can see in Fig 4, the recognition rate is close to 100% under the improved CS-LSSVM algorithm, which illustrates the effectiveness of the newly proposed algorithm. Compared with other intelligent algorithms, the proposed algorithm in this paper can improve the recognition accuracy greatly, refer to Table 2 for more details.

**Table 2:** The fault pattern recognition results based on different methods.

Algorithm	Number of Samples	*C*	*σ*	Precision
PSO-LSSVM	35	6.705	18.675	85.71%
GA-LSSVM	35	13.067	9.7103	91.43%
CS-LSSVM	35	17.717	6.5610	94.29%
ICS-LSSVM	35	12.373	8.6546	99.96%

## 5. Conclusions

For LSSVM, precise selection of the parameter in RBF kernel function and the penalty factor is the key to recognize the fault pattern. Since the artificial parameter setting may lead to inaccurate recognition, an improved CS algorithm has been proposed in this paper to optimize parameters. The simulation results have illustrated that the improved CS algorithm can converge to the global optimum quickly, and lead to perfect fault pattern recognition performance of ship power equipments. The improved CS-LSSVM can provide a technical support for maintaining ship power equipments timely and accurately.
